# *Pseudohampsonella* : a new genus of Limacodidae (Lepidoptera: Zygaenoidea) from China, and three new species

**DOI:** 10.1093/jis/14.1.46

**Published:** 2014-01-01

**Authors:** Alexey V. Solovyev, Aidas Saldaitis

**Affiliations:** 1 Ulyanovsk State Pedagogical University, 100-letiya Lenina sq. 4, RUS-432700, Ulyanovsk, Russia; 2 Nature Research Centre, Akademijos str. 2, LT–08412 Vilnius-21, Lithuania

**Keywords:** Indomalaya

## Abstract

A new genus, *Pseudohampsonella* gen. n. (typespecies: *Pseudohampsonella erlanga* Solovyev & Saldaitis), and three new species, *Pseudohampsonella erlanga* sp. n. Solovyev & Saldaitis (from Sichuan Province, China), *Pseudohampsonella hoenei* sp. n. Solovyev & Saldaitis (Yunnan Province, China), and *Pseudohampsonella argenta* sp. n. Solovyev & Saldaitis (Yunnan Province, China) are described. The taxonomic position of the genus is discussed.

## Introduction

During ongoing Southeast Asian Limacodidae research, three new species that cannot be attributed to any known limacodid genus because of the modified external and genital characters ( Figures 1–5 , 10–23 ) were recognized. Externally, these species somewhat resemble some species of *Hampsonella* Dyar, 1898 (typespecies: *Parasa dentata* Hampson, 1893) ( Figures 6 , 7 ), *Caissa* Hering, 1931 (typespecies: *C. caissa* Hering, 1931) ( Figure 8 ), and *Pseudocaissa*Solovyev & Witt, 2009 (typespecies: *P* . *apiata*Solovyev & Witt, 2009 ) ( Figure 9 ), but morphologically are quite different from all of them, showing a high level of morphological modifications that cannot be associated with any limacodid genus, so a new genus is proposed for them here.

**Figures 1–9.**
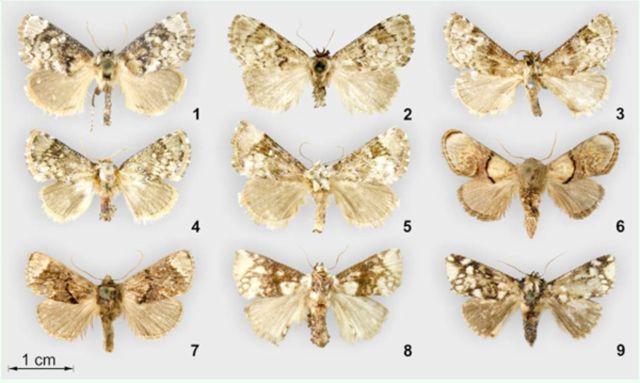
External view of *Pseudohampsonella***gen. n.** and related genera; **1** – *Pseudohampsonella erlanga***sp. n.** , holotype, ♂ (in MWM); **2** – *P. erlanga***sp. n.** , holotype, ♀ (in MWM); **3** – *P. hoenei***sp. n.** , holotype, ♂ (in ZFMK); **4** – *P. argenta***sp. n.** , holotype, ♂ (in MWM); **5** – *P. argenta***sp. n.** , paratype, ♀ (in MWM); **6** – *Hampsonella dentata* (Hampson, 1893), N.E. India, ♂ (in MWM); **7** – *H. arizana* (Wileman, 1916), Taiwan, ♂ (in MWM); **8** – *Caissa caissa* Hering, 1931, holotype, India, ♂ (in BMNH); **9** – *Pseudocaissa apiata*Solovyev & Witt, 2009 , holotype, northern Vietnam, ♂ (in MWM). High quality figures are available online.

**Figures 10–11.**
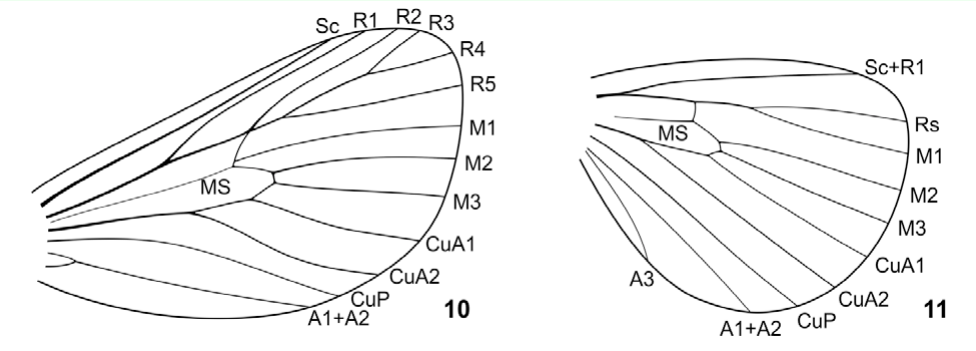
Wing venation of *Pseudohampsonella erlanga***sp. n** .; **10** – forewing; **11** – hindwing. High quality figures are available online.

**Figures 12–23.**
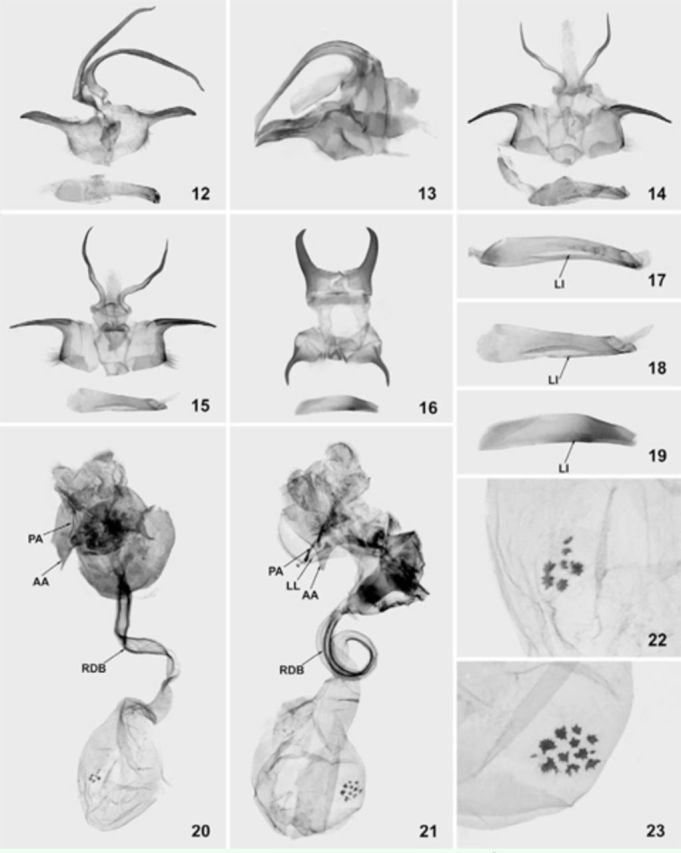
Genitalia of *Pseudohampsonella***gen. n.** ; **12** – *P. erlanga***sp. n.** , holotype, ♂ (in MWM); **13** – *P. erlanga***sp. n.** , paratype, lateral view, ♂ (in AFM); **14** – *P. hoenei***sp. n.** , holotype, ♂ (in ZFMK); **15** – *P. hoenei***sp. n.** , paratype, ♂ (in AFM); **16** – *P. argenta***sp. n.** , holotype, ♂ (in MWM); **17** – enlarged aedeagus of *P. erlanga***sp. n.** , paratype, ♂ (AFM); **18** – enlarged aedeagus of *P. hoenei***sp. n.** , paratype, ♂ (in AFM); **19** – enlarged aedeagus of *P. argenta***sp. n.** , holotype, ♂ (in MWM); **20** – *P. erlanga***sp. n.** , paratype, ♀ (in MWM); **21** – *P. argenta***sp. n.** , paratype, ♀ (in MWM); **22** – enlarged signum of corpus bursae of *P. erlanga***sp. n.** , paratype, ♀ (in MWM); **23** – enlarged signum of corpus bursae of *P. argenta***sp. n.** , paratype, ♀ (in MWM). Abbreviations: *AA* – anterior apophysis; *LI* – longitudinal incision of aedeagus; *LL* – lateral lobe; *PA* – posterior apophysis; *RDB* – sclerotized longitudinal ribbons in ductus bursae. High quality figures are available online.

## Materials and Methods

The material examined was collected during June and July 1935; July 1937; May, June and July 1999; July 2004; July 2009; and August 2011, using artificial light. Taxonomic nomenclature used in this study was developed with reference to taxonomical experts and relevant literature ( Holloway 1986 ; Holloway et al. 1987 ; Epstein 1988 , 1996; Solovyev and Witt 2009 ; Wu and Fang 2008 ; Wu and Fang 2009 ).

Digital images were made using a Nikon ( www.nikon.com ) Coolpix 5400 camera, a Canon ( www.canon.com ) EOS 450D camera with reversed lens Canon EF-S 18–55 mm and a binocular microscope MBS-9. The images were improved and prepared for publication using Corel Draw 15 and Corel Photo-Paint 15 ( www.corel.com ).

The genitalia of both sexes were examined using standard methods. Abdomens were macerated in a heated 10% aqueous alkali solution for 10 minutes before genitalia were dissected with a micro-forceps. The separated aedeagus and complete female genitalia were stained with Evans blue dye (0.1% aqueous) for five minutes prior to being mounted in Euparal and labeled.

Molecular analyses of two specimens were performed at the Canadian Centre for DNA Barcoding, University of Guelph, under the project LIMBC of BOLD ( www.boldsystems.org ). The sequences were aligned using Mega 5 with integrated ClustalW ( www.ebi.ac.uk/Tools/msa/clustalw2 ). The pairwise distances were calculated in Mega 5 ( www.megasoftware.net ) ( Tamura et al. 2011 ) using Distance Estimation analysis with the following criteria: transitions and transversions included, all positions of codons are used, and number of differences model.

### Nomenclature

This publication and the nomenclature it contains have been registered in ZooBank. The LSID number is:

urn:lsid:zoobank.org:pub:23E8ACD1-60E2-4971-9275-3656B60D86B5 .

It can be found online by inserting the LSID number after www.zoobank.org/ .

### List of Abbreviations

AFM: Alessandro Floriani – Milan, Italy

BOLD: The Barcode of Life Data System ( www.boldsystems.org )

BMNH: Natural History Museum – London, United Kingdom

CAS: research collection of Alexey V. Solovyev – Ulyanovsk, Russia

LIMBC: a project within BOLD, ‘DNA barcoding Limacodidae moths’

MWM: Museum Witt – Munich, Germany

NRCV: Nature Research Centre – Vilnius, Lithuania

ZFMK: Zoologisches Forschungsmuseum Alexander Koenig – Bonn, Germany

### Systematic accounts

*Pseudohampsonella* **Solovyev & Saldaitis, gen. n.**

Type species: *Pseudohampsonella erlanga***sp.n** . designated herein.

( Figures 1–5 , 10–23 )

**Male** ( Figures 1 , 3 , 4 , 10 , 11 ): The forewing costal margin length is 9.5–13.0 mm. The antennae are filiform, serrate, and somewhat flattened. The galea is short, as long as the first segment of the labial palp or slightly longer. The prothorax is brown; remainder of thorax is whitish grey with sparse dark brown scales. The forewing is ochre brown with a speckled spotty pattern including zigzag-like, weakly defined dark brown antemedial fascia bordered distally by diffused white or silver scales, postmedial and subterminal fasciae, and a terminal pale area; the postmedial fascia consists of rounded whitish spots, spot in discal area is usually distinct; the forewing pale terminal area reaches proximally 3/4 of the costa almost to the tornus, with a characteristic small brown streak near the costa; the forewing fringe is brown, with tufts of white scales; the hindwings are ochre brown, with diffuse brown streak near the tornus, with pale terminal wing area and pale fringe; abdomen ochre brown. The hind tibia bears four spurs. In forewing venation, the vein R1 is curved; R3+R4 form a common stem; the vein R5 is branched from R3+R4; the medial stem is undivided; the vein CuA2 is curved ( Figure 10 ). In hindwing, the vein Sc+R1 and radial stem are anastomosed basally; the medial stem is undivided; the veins Rs and M1 form a long common stem ( Figure 11 ); the frenulum is developed.

**Female** ( Figures 2 , 5 ): The forewing costal margin length is about 13.0 mm. The antennae are filiform, not serrate. The females are almost undistinguishable from the males by the forewing pattern and coloration, but usually somewhat dull.

**Male genitalia** ( Figures 12–19 ): Strongly modified; the uncus is long, deeply divided into a pair of pincer-like lobes. The gnathos is reduced and represented by weak, sclerotized tape; the tuba analis is tubular, sclerotized ventrally. The juxta is small, triangle-like. The valvae are broad proximally and very slender, horn-like distally, with chaeta in tornus. The saccus is short. The aedeagus is almost straight, gradually curved, with longitudinal incision in lateral surface in distal 1/3–2/3 of aedeagus ( Figures 17–19 : *LI* ).

**Female genitalia** ( Figures 20–23 ): The ovipositor lobes are flattened, somewhat elongated; the 8 ^th^ abdominal segment with elongated, finger-shaped, membranous lateral lobes ( Figure 21 : *LL* ); the vaginal plate is spinulate. The antrum is broad, well developed, and strongly sclerotized. The ductus bursae is slightly spiraled, with two medial, slender, strongly sclerotized, longitudinal ribbons ( Figures 20 , 21 : *RDB* ). The corpus bursae is rounded, with small area of stellate signa ( Figures 22 , 23 ).

**Diagnosis** : The new genus is well distinguished externally from all known Limacodidae by the modified spotty wing pattern, though this bears some resemblance to that of species of *Caissa* and *Pseudocaissa.* However, the antemedial fascia of *Pseudohampsonella* is different, irregularly dentate throughout, with a more prominent indentation at one third from the dorsum (it is straight in *Caissa* and *Pseudocaissa* ); the compact pale spot is forever developed in the forewing discal area of *Pseudohampsonella* . The compact, small dark spot situated near the hindwing tornus that is highly diagnostic for *Caissa* and *Pseudocaissa* ( Figures 8 , 9 ) is absent in *Pseudohampsonella* . Several members of the genus *Hampsonella* (e.g. *H. arizana* (Wileman, 1916), Figure 7 ) are found to be externally similar to *Pseudohampsonella* by the speckled forewing pattern and dentate antemedial fascia, but the pattern in *Pseudohampsonella* is spotty, and the antemedial fascia has a characteristic prominent dent in the lower part. The male genitalia of *Pseudohampsonella* are mostly diagnostic and extremely modified, unusually within the Limacodidae. The uncus is divided into two horn-like lateral lobes ( Figures 12–16 ), the gnathos is reduced to a small tape, the tuba analis is sclerotized ventrally, the valvae are broad proximally and very slender, horn-like distally, without saccular processes, and the aedeagus is dissected (incised) laterally in the distal part ( Figures 17–19 ). The female genitalia are unlikely for *Caissa* and *Hampsonella* characterized by two well developed and strongly sclerotized longitudinal ribbons in the ductus bursae.

**Species composition:** *P. erlanga* **sp. n** ., *P. hoenei***sp. n** ., and *P. argenta***sp. n.**

**Bionomics:** All species included in the genus are known from China at elevations of 2100– 4000 m a.s.l., univoltine. The biology of its members requires additional study. Immature stages are unknown.

**Molecular data:** The data was obtained for two species, *P. erlanga* sp. n. (LIMBC391-11) and *P. hoenei* sp. n. (LIMBC390-11), and consists of a fragment of COI-5P with a length of 658 bp, which is used in DNA-barcoding of species ( Hebert et al. 2003 ). The two species differ by 6.4% (see Appendix for full data).

**Etymology** : The genus is named in connection with its similarity to the genus *Hampsonella* , using the prefix “pseudo” meaning “false” in Latin.

*Pseudohampsonella erlanga* **Solovyev & Saldaitis, sp. n.**

( Figures 1 , 2 , 12 , 13 , 17 , 20 , 22 )

**Male** ( Figures 1 , 10 , 11 ): The forewing length is ca. 10.0–13.0 mm. General characteristics are as in the generic account. The thorax is white, with sparse dark brown scales, and a dark brown prothorax. The forewing pattern is typical for the genus; the postmedial fascia bears white rounded spots and white discal spot; the forewing fringe is brown, with rare tufts of white scales; the hindwings are ochre brown, with yellowish fringe and diffuse brown streak near the tornus.

**Female** ( Figure 2 ): The forewing length is ca. 14.0 mm. The antennae are filiform, not serrate. The females are almost not distinguishable from the males by the forewing pattern and coloration, however somewhat dull.

**Male genitalia** ( Figures 12 , 13 , 17 ): The uncus is bilobed, longer than the valva; each lobe is very slender, horn-shaped, curved, and long ( Figures 12–13 ). The gnathos is reduced and represented by the sclerotized tape connecting bases of uncus lobes. The valvae are widened proximally, very slender distally, and somewhat claw-shaped apically; the tornus of the valvae is covered with long chaeta. The juxta is triangle-shaped with deep medial incision. The saccus is short. The aedeagus is slightly curved, somewhat longer than the valvae, tubular, with deep longitudinal incision on the lateral surface running from the aedeagus apex to its basal 1/3 ( Figure 17 ).

**Female genitalia** ( Figures 20 , 22 ): The ovipositor lobes are flattened, ovoid ( Figure 20 ). The anterior apophyses are short, triangular ( Figure 20 : AA); the posterior apophyses are long and slender ( Figure 20 : PA). The vaginal plate is spinulate. The antrum is short, strongly sclerotized. The ductus bursae is slightly spiraled, with pair of medial strongly sclerotized longitudinal ribbons. The corpus bursae is ovoid, with small area of stellate signa ( Figure 22 ).

**Holotype:** ♂ ( Figure 1 ), ‘China / Sichuan | Erlangshan Mts. | E Luding, 2560 m | 19– 23.VII.2004 | leg. S Murzin | Museum Witt’ (in MWM, slide 11472).

**Paratypes:** 4♂♂, as holotype (in MWM, slide 12318); 2♂♂, 1♀, China, Sichuan, Daxue Shan Mts (S), 80 km W Mianning, 28º34’N, 102ºE, 2750 m, 7–8.VII.1999, leg. Sinaev & Plutenko (in MWM, slides 17926 (♂), 20296 (♀)); 1♂, China, Sichuan, Daxue Shan Mts (S), 80 km W Mianning, 28º34’N, 102ºE, 7– 8.VII.1999, leg. Sinaev & Plutenko (in MWM); 5♂♂, China, W. Sichuan, Kangding evn. 3000 m, 29º53’N, 101º55’E, 13.VII.2009, leg. I. & A. Floriani (in AFM, NRCV, slides 10-08, 10-14); 1♂, as previous (in MWM); 1♂, China, W. Sichuan, road Ya’an / Kangding, Erlang Shan Mt., 29º51’N, 102º18’E, 2100 m, 12.VII.2009, leg. I. & A. Floriani (in AFM); 1♂, as previous (in MWM); 1♂, China, W. Sichuan, road Yaan / Kangding, Erlang Shan Mt., 29º87.340’N, 102º30.970’E, 2200 m, 02.VIII.2011, leg. A. Floriani (in AFM, slide LIMAC-12-01).

**Diagnosis** : Externally the species clearly differs from *P. argenta* sp. n. ( Figures 4 , 5 ) by the absence of silver scales on forewings and well-expressed antemedial fascia, and from *P. hoenei* sp. n. ( Figure 3 ) by its rounded white spots forming postmedial fascia. The male genitalia are diagnostic. They are somewhat similar to *P. hoenei* sp. n. ( Figures 14 , 15 ) as the small base of the uncus bears very slender horn-like lobes (the width of the lobe is less than half the width of the aedeagus). *P. erlanga* sp. n. is distinguished from *P. hoenei* sp. n. by the uncus lobes which are distinctly longer than the valvae, the proximal part of the valvae being more ovoid, not square-shaped, without distinct tornal corners and the distal part of the valvae are not sickle-shaped. The female genitalia are distinguished from those of *P. argenta* sp. n. ( Figure 21 ) by longer ductus bursae, smaller field of stellate signa in corpus bursae, shorter antrum. The females of the rest congener, *P. hoenei* sp.n., are unknown.

**Bionomics and distribution:** Only known from one generation in the Erlang Shan and Daxue Shan Mountains in the Kangding area of China’s Sichuan Province on the east edge of the Tibetan plateau. Most specimens were collected in July at elevations ranging from 2100 to 3000 m a.s.l. in a mountainous virgin mixed forest habitat dominated by various broad-leaved trees such as oaks ( *Quercus dentata* , *Q. glauca* ), poplars ( *Populus cathayana* , *P. simonii* ),elm ( *Ulmus parvifolia* ), rho-dodendrons ( *Rhododendron brachycarpum* , *R. dauricum* ), and bamboos ( *Phyllostachys* spp., *Borinda* spp., *Fargesia* spp.).

**Molecular data:** See generic description and Appendix.

**Etymology:** The name is toponomical.

*Pseudohampsonella hoenei* **Solovyev & Saldaitis, sp. n.**

( Figures 3 , 14 , 15 , 18 )

**Male** ( Figure 3 ) : The forewing length is ca. 10.0–12.5 mm. General characteristics are as given in the description of the genus. The thorax is yellowish–pale-gray, with sparse dark brown scales; the tegulae are dark brown apically. The forewings' antemedial fascia is dark brown, with external border of whitish grey scales; the postmedial fascia is dentate, without distinct rounded spots; the terminal forewing area is pale, with proximal border running from 3/4 costa to the tornus, with dark brown streak on the costa; the forewing fringe is brown, with sparse tufts of white scales.

**Female:** Unknown.

**Male genitalia** ( Figures 14 , 15 , 18 ): The uncus is bilobed, horn-shaped, and similar in length to the valva; each lobe is very slender and irregularily curved ( Figures 14 , 15 ). The gnathos is reduced to sclerotized tape connecting both lobes of the uncus. The juxta is triangle-shaped with deep medial incision. The valvae are square-shaped proximally, with distinct tornal corner covered with long chaeta and sickle-shaped, very narrow distally. The saccus is short. The aedeagus is slightly curved, with longitudinal incision running from aedeagus apex to basal third ( Figure 18 ).

**Holotype:** ♂( Figure 3 ), [China, Yunnan] ‘Li-kiang ca. 3000 m | Prov. Nord-Yuennan | 4.7.1935. H. Höne’ (in ZFMK, slide 10-15).

**Paratypes:** 1♂, as holotype, but, 4000 m, 27.VI.1935 (in ZFMK); 1♂, as holotype, but 4000 m, 4.VII.1935 (in ZFMK); 1♂, as holotype, but 3000 m, 6.VII.1935 (in ZFMK); 1♂, as holotype, but 4000 m, 29.VI.1935 (in ZFMK); 1♂, A-tun-tse (N. Yuennan), ca. 4000m, 24.VII.1937, H. Höne (in ZFMK); 1♂, Li-Kiang, (China), Provinz Nord-Yuennan, 21.VI.1935, H. Höne (in MWM, slide 17927); 3♂♂, China, N. Yunnan, road Xinngcheng / Zhongdian, 28º32’N, 99º49’E, 3500 m, 18.VII.2009, leg. I. & A. Floriani (in AFM, NRCV, slides 10-09; 10-12); 2♂♂, as previous (in MWM); 6♂♂, China, N. Yunnan, road Zhongdian / Lijiang, 27º28’N, 99º53’E, 3000 m, 19.VII.2009, leg. I. & A. Floriani (in AFM, NRCV).

**Diagnosis:** Externally the species is distinguished from the rest of its congeners by the dentate postmedial fascia, which is without rounded whitish spots, and from *P. argenta***sp. n.** ( Figure 4 ) by the absence of silver scales on forewings and dark brown antemedial fascia. The male genitalia are similar to those of *P. erlanga* sp. n. ( Figures 12 , 13 ), as both species have an uncus with a smaller base and narrow uncus lobes, but in *P. hoenei***sp. n** . the length of the lobes of the uncus is similar in the length to the valva, the base of valva is rather square-shaped, with a distinct tornal corner, not ovoid as in *P. erlanga***sp. n** .

**Bionomics and distribution:** Only known from a few localities in the northern part of Yunnan Province on the east edge of the Tibetan plateau. This high mountain new species was collected in late June and throughout July at elevations ranging from 3000 to 4000 m a.s.l. in virgin mixed forest with swampy and mossy meadows. The habitat is dominated by various species of *Alnus* , *Prunus* , *Quercus* , *Rhododendron* , *Abies* , different species of small bamboos, and other smaller shrubs and ferns.

**Molecular data:** See generic description and Appendix.

**Etymology:** The species is named after Mr. H. Höne, the collector of the holotype, and several paratypes of this species.

*Pseudohampsonella argenta* **Solovyev & Saldaitis, sp. n** .

( Figures 4 , 5 , 16 , 19 , 21 , 23 )

**Male** ( Figure 4 ): The forewing length is ca. 9.5–10.5 mm. The thorax is yellowish pale grey, with sparse dark brown scales. The forewing basal area is not well expressed. The antemedial fascia is not well developed, zigzag, dark brown, with sparse silver scales. The postmedial fascia consists of a series of ochre rounded spots; the discal spot is rounded, yellowish grey. The terminal forewing area is grey, with proximal border running from 3/4 costa almost to the tornus (approximately to the vein CuA1), with dark brown streak on costa. The forewings have silver spots in the region of terminal fascia; forewing fringe is brown, with rare tufts of white scales.

**Female** ( Figure 5 ): The forewing length is ca. 13.0 mm. The antennae are filiform, not serrate. The females are almost not distinguished from the males by the forewing pattern and coloration, however they are slightly duller.

**Male genitalia** ( Figures 16 , 19 ): The uncus bilobed, with wide base, pincers-like, curved, longer than the valva, horn-shaped, strongly sclerotized, and very robust; basal width of each lobe is similar to aedeagus in width ( Figure 16 ). The gnathos is reduced to a weakly developed sclerotized tape; the tuba analis is sclerotized ventrally. The valvae are weakly sclerotized, wide and rounded in proximal half, and strongly sclerotized, very narrow and acute apically, claw-shaped in its distal half. The juxta is small, with deep medial incision. The aedeagus is tubular, slightly curved, with longitudinal deep incision in lateral surface, running from the aedeagus apex to distal two-thirds ( Figure 19 ).

**Female genitalia** ( Figures 21 , 23 ): The ovipositor lobes are flattened, ovoid ( Figure 21 ). The posterior apophyses are long and slender ( Figure 21 : PA); the anterior apophyses are short, triangular ( Figure 21 : AA). The vaginal plate is spinulate. The 8 ^th^ abdominal segment has elongated, finger-shaped, membranous lateral lobes ( Figure 21 : LL). The antrum is strongly sclerotized, wide. The ductus bursae is slightly spiraled, with a pair of medial strongly sclerotized longitudinal ribbons. The corpus bursae is ovoid, with small area of stellate signa ( Figure 23 ).

**Holotype:** ♂( Figure 4 ), ‘China / Yunnan – prov. (NW) | Dali Bai autonom. pref.; Yunlong | county; Fengshuining – Mts., 2460 m | ------13 km N Coajian ----| 10–20.V.1999; 25, 46ºN / 99, 06ºE | leg. / ex. coll. Dr. R. Brechlin | Museum Witt’ (in MWM, slide 12332) [‘Coajian’ – original misprint in the holotype label, should be read as ‘Caojian’].

**Paratypes:** 1♂, as holotype; 3♂♂, 1♀, China, Yunnan, 13 km N Caojian, Fengshuining Mts., Yunlong, 25º46’N, 99º06’N, 2460 m, 20.V–9.VI.1999, leg. local collector (in MWM, slides 11484 (♂) & 12317 (♀)); 1♂, China, Yunnan, 13 km N Caojian, Fengshuining Mts., 25º46’N, 99º06’E, 2460 m, 10– 20.V.1999, leg. Dr. Brechlin (in MWM); 1♂, China, Yunnan, 13 km N Caojian, Yunlong, Fengsuining Mts, 2460 m, 20.V–9.VI.1999, leg Dr. Ronald Brechlin (in MWM).

**Diagnosis:** Externally the species is distinguished from its congeners ( Figures 1–3 ) by the presence of silver scales in the antemedial and terminal fasciae and poorly-expressed proximal area of the forewings and brown antemedial fascia. The male genitalia clearly distinguish this species ( Figure 16 ); uncus very robust, with large and wide base (width of uncus is diagnostically much larger than basal width of valva) and wide horn-like lobes (their width is similar to that of aedeagus); base of the valva rather rounded; distal part of the valva strongly sclerotized and claw-shaped. The female genitalia are somewhat different from those of *P. erlanga* sp. n *.* ( Figure 20 ); the species *P. argenta* sp. n. is characterized by longer antrum, shorter ductus bursae, larger area of stellate signa in corpus bursae; unfortunately females of *P. hoenei* sp. n. are unknown, and their diagnostic characters in female genitalia are not known.

**Bionomics and distribution:** Only known from a few localities in China’s Yunnan Province on the east edge of the Tibetan plateau. It is a mountain species (known only from the altitude of 2460 m a.s.l.), with adults observed from mid-May to early June.

**Etymology:** The species name derives from the Latin word “argentum” for silver, which is the color of the forewing scales unique to members of the genus.

## Discussion

**Figures 24–36.**
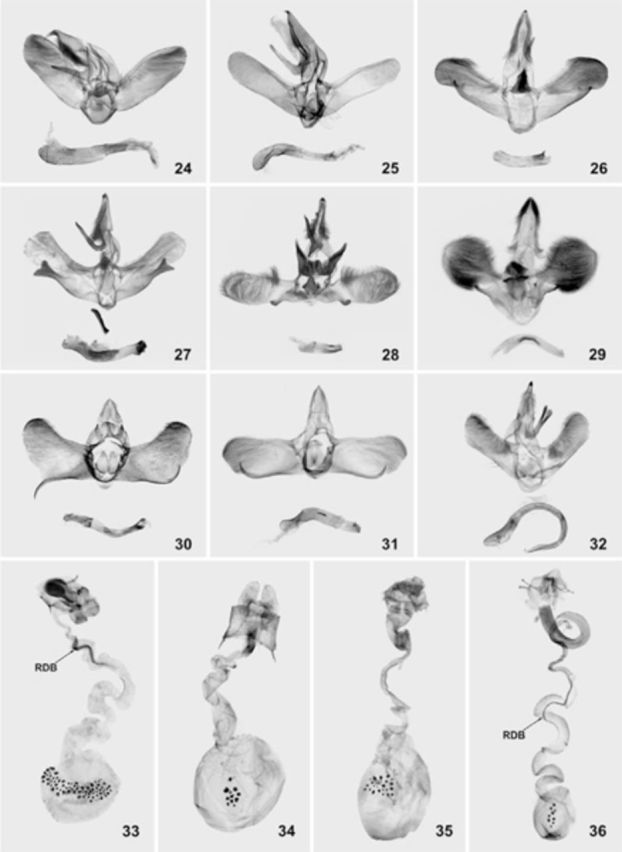
Genitalia of some members of stellate-signa limacodid group; **24** – *Hampsonella dentata* (Hampson, 1893), ♂, India, syntype (in BMNH); **25** – *H. arizana* (Wileman, 1916), ♂, Taiwan, syntype (in BMNH); **26** – *Caissa caissa* Hering, 1931, holotype, ♂, India, (in BMNH); **27** – *C. aurea*Solovyev & Witt, 2009 , holotype, ♂, northern Vietnam (in MWM); **28** – *C. parenti* Orhant, 2000, ♂, central Vietnam (in CAS); **29** – *Pseudocaissa apiata*Solovyev & Witt, 2009 , holotype, ♂, northern Vietnam, ♂ (in MWM); **30** – *Apoda limacodes* (Hufnagel, 1766), ♂, Ukraine (in MWM); **31** – *A. y-inversum* (Packard, 1864), ♂, USA, Undiana (in CAS); **32** – *Ceratonema butleri* Kawada, 1930, ♂, Japan: Yamanashi (in MWM); **33** – *Hampsonella dentata* (Hampson, 1893), ♀,northern Vietnam (in MWM); **34** – *H. membra*Solovyev & Witt, 2009 , ♀, northern Vietnam (in CAS); **35** – *Caissa fasciatum* (Hampson, 1893), ♀, northern Myanmar (in MWM); **36** – *Apoda y-inversum* (Packard, 1864), ♀, USA: Indiana (in CAS); Abbreviation: *RDB* – sclerotized longitudinal ribbons in ductus bursae. High quality figures are available online.

The genus *Pseudohampsonella* gen. n. can be associated with the stellate-signa group (sensu Holloway 1986 ; Holloway et al. 1987 ) or *Apoda* group (sensu Epstein 1988 , 1996); these groups were designated independently for Old World and New World taxa respectively, but together are probably monophyletic. The stellate-signa group includes the genera *Altha* Walker, 1862; *Apoda* Haworth, 1809; *Ara-bessa*Solovyev & Witt, 2009 ; *Atosia* Snellen, 1900; *Austrapoda* Inoue, 1982, *Barabashka*Solovyev & Witt, 2009 ; *Belippa* Walker, 1865; *Caissa* Hering, 1931; *Ceratonema* Hampson, 1893; *Chalcocelis* Hampson, 1893; *Chalcoscelides* Hering, 1931; *Cheromettia* Moore, 1883; *Demonarosa* Matsumura, 1931; *Fignya*Solovyev & Witt, 2009 ; *Flavinarosa*Holloway, 1986 ; *Hampsonella* Dyar, 1898; *Heringarosa*Holloway, 1986 ; *Heterogenea* Knoch, 1783; *Hoyosia* Agenjo, 1972; *Kitano-la* Matsumura, 1925 ; *Mediocampa* Inoue, 1982; *Nagoda* Moore, 1887; *Narosa* Walker, 1855 s. str. (including subgenus *Penicil-lonarosa* Strand, 1916); *Pectinarosa*Holloway, 1987 ; *Pseudocaissa*Solovyev & Witt, 2009 ; *Quasinarosa*Solovyev & Witt, 2009 ; *Saccurosa*Holloway, 1986 ; *Sansarea*Solovyev & Witt, 2009 ; and *Trichogyia* Hampson, 1894. Some of their species are illustrated on Figures 6–9 and 22–34 , and their morphology is already described in Holloway 1986 ; Holloway et al. 1987 ; Solovyev and Witt 2009 ; Wu and Fang 2008 ; and Wu and Fang 2009 . The *Apoda* group includes the genera *Apoda* Haworth, 1809 ( Figures 30 , 31 , 36 ); *Heterogenea* Knoch, 1783; *Lithacodes* Packard, 1864; *Packardia* Grote & Robinson, 1866; and *Tortricidia* Packard, 1864 ( Epstein 1996 ).

The morphology of both stellate-signa and *Apoda* groups is characterized by the presence of an elongate, somewhat elliptical field of small, rounded, stellate-spined signa in female corpus bursae ( Figures 33–36 ), which is also found in *Pseudohampsonella* ( Figures 22 , 23 ). However, the *Apoda* group is characterized by the veins Rs and M1 in the hindwings separated by a perpendicular or oblique cross vein at the end of the discal cell ( Epstein 1996 ); on the contrary, in *Pseudohampsonella* (as in many other members of stellate-signa group, including *Caissa* and *Hampsonella* ), the veins Rs and M1 in the hindwing form a stalk. The male antennae of the members of both groups are usually filiform. In most of the genera the forewings bear transverse antemedial and subterminal fasciae (igures 6–9), forming an “arcuate” pattern; however, it is distinctly de-viant in *Pseudohampsonella* and remotely similar to that of some members of *Caissa, Pseudocaissa* , and *Hampsonella* , as it noted in generic diagnosis; *Pseudohampsonella* differs from the latter by a rather spotted forewing pattern, irregularly dentate antemedial fasciae bearing a large prominent dent in the lower part, with compact pale spot in the discal area.

The male genitalia of stellate-signa and *Apoda* groups are diverse ( Figures 24–32 ); however, they are usually only slightly modified in comparison with other most Limacodidae, with uncus entire, gnathos only very rarely reduced, simple, elongate valvae without spur-like distal portion; aedeagus is without large lateral incision as in *Pseudohampsonella* . The male genitalia of *Pseudohampsonella* are quite different from all known members of the groups and worldwide Limacodidae, with uncus divided into two horn-like lobes, short and spur-like distally valvae, aedeagus with large longitudinal incision. The ductus bursae in the female genitalia of *Pseudohampsonella***gen. n.** has a pair of medial, longitudinal, and strongly sclerotized ribbons (most other members of the stellate-signa and *Apoda* groups usually have at least only one ribbon or the ribbon is not developed). The larvae of the groups are smooth, without scoli. Most probably the larva of *Pseudohampsonella***gen. n.** is also smooth.

Thus, the genus *Psudohampsonella***gen. n.** represents a particularly strongly modified lineage within the stellate-signa and *Apoda* groups, and indeed within the Limacodidae generally.

## Appendix

**Appendix**

The sequences of COI-5P (PCR primers: LepF1/LepR1) of *Pseudohampsonella* spp.:

*Pseudohampsonella erlanga* **sp. n.** (barcode ID in BOLD: LIMBC391-11; museum ID: WITT-LIMAC-025 (the specimen is kept in NRCV)):

AACTTTATATTTTATTTTCGGAATTTGA GCAGGAATAGTAGGTACATCTTTAAGT TTGATAATTCGAGCAGAATTAGGAAAC CCAGGATCTTTAATTGGTGATGATCAA ATTTATAATACTATTGTCACTGCTCATG CTTTTATTATAATTTTTTTTATAGTTAT ACCTATTATAATTGGAGGATTTGGTAA TTGATTAGTACCTTTAATATTGGGAGC CCCTGATATAGCTTTCCCACGAATAAA TAATATAAGATTTTGATTATTACCTCCT TCTCTTATATTACTTATTTCCAGTAGAA TTGTAGAAAACGGGGCAGGAACAGGG TGAACAGTATACCCGCCCCTCTCTTCT AATATTGCCCACAGTGGAAGATCAGTA GATTTAGCAATTTTTTCTCTTCATTTAG CTGGAATTTCATCAATTTTAGGGGCTG TAAATTTTATTACAACTATTATTAATAT ACGTCCTAATGGAATATCATTTGATCA AATACCTTTATTTGTTTGAGCTGTAGG AATTACAGCCCTATTACTTCTCCTCTCT CTACCTGTTTTAGCAGGAGCTATTACT ATATTACTTACTGACCGAAATTTAAAT ACTTCTTTTTTCGACCCAGCAGGAGGG GGAGATCCAATTCTCTACCAACACTTA TTT

*Pseudohampsonella hoenei* sp. n. (barcode ID in BOLD: LIMBC390-11; museum ID: WITT-LIMAC-024 (the specimen is kept in NRCV)):

AACTTTATACTTTATTTTTGGAATTTGA GCAGGAATAGTAGGTACATCTTTAAGT TTAATAATTCGAGCAGAATTAGGAAAT CCTGGATCTTTAATTGGTGATGATCAA ATTTATAATACTATTGTTACAGCTCATG CTTTTATTATAATTTTTTTTATAGTTAT ACCTATTATAATTGGAGGATTTGGTAA TTGATTAGTACCTTTAATACTAGGAGC TCCTGATATAGCTTTCCCACGAATAAA TAATATAAGATTTTGATTATTACCCCCC TCTCTTATACTACTTATTTCTAGAAGTA TTGTAGAAAATGGGGCAGGAACAGGT TGAACAGTGTATCCACCTTTATCTTCA AACATTGCTCATAGAGGAAGATCAGTA GATTTAGCAATTTTTTCTCTTCATTTAG CTGGAATTTCATCAATCTTAGGGGCTG TAAATTTTATTACAACTATTATTAATAT ACGTCCTAATGGAATATCATTTGATCA AATACCTTTATTTGTTTGAGCTGTAGG AATTACAGCCTTATTACTTCTTCTTTCT TTACCTGTTTTAGCAGGAGCTATTACT ATATTATTAACTGACCGGAATTTAAAT ACTTCTTTTTTTGACCCTGCAGGAGGG GGAGATCCTATTCTTTATCAACACTTAT TT
